# Supplementary material for: Leg Dominance Effects on Postural Control When Performing Challenging Balance Exercises
Source: Brain Sci. 2020 Feb 25;10(3):128. doi: 10.3390/brainsci10030128 (PMC7139434; doi:10.3390/brainsci10030128)
Supplement: Supplementary file 1 [file brainsci-10-00128-s001.zip › Supplementary Table 1.docx]

**Table S1.** Repeated all analysis procedures with several cut-off frequencies (in the range 3-13 Hz) to ensure the presented results (the number of zero-crossings and the standard deviation of time between zero-crossings) which were non-coincidental and not influenced by the filtering artifacts. The number “1” represents significant difference at *p* < 0.05. PM is principal movements. The asterisk represents the cut-off frequency (10 Hz) used in the current study.

| **Number of zero-crossings** | | | | | | | | |
| --- | --- | --- | --- | --- | --- | --- | --- | --- |
| Hz | PM 1 | PM 2 | PM 3 | PM 4 | PM 5 | PM 6 | PM 7 | PM 8 |
| 3 | 0 | 0 | 0 | 0 | 0 | 0 | 0 | 0 |
| 4 | 1 | 0 | 0 | 0 | 0 | 0 | 0 | 0 |
| 5 | 0 | 0 | 0 | 0 | 0 | 0 | 0 | 0 |
| 6 | 0 | 0 | 0 | 0 | 0 | 1 | 0 | 0 |
| 7 | 0 | 0 | 1 | 0 | 0 | 1 | 0 | 0 |
| 8 | 0 | 0 | 1 | 0 | 0 | 1 | 0 | 0 |
| 9 | 0 | 0 | 1 | 0 | 0 | 1 | 0 | 0 |
| 10* | 0 | 0 | 1 | 0 | 0 | 1 | 0 | 0 |
| 11 | 0 | 0 | 1 | 0 | 0 | 1 | 0 | 0 |
| 12 | 0 | 0 | 1 | 1 | 0 | 1 | 0 | 0 |
| 13 | 0 | 0 | 1 | 1 | 0 | 1 | 0 | 0 |
| **Standard deviation of time between zero-crossings** | | | | | | | | |
| Hz | PM 1 | PM 2 | PM 3 | PM 4 | PM 5 | PM 6 | PM 7 | PM 8 |
| 3 | 0 | 0 | 1 | 0 | 0 | 0 | 0 | 0 |
| 4 | 0 | 0 | 1 | 0 | 0 | 0 | 0 | 0 |
| 5 | 0 | 0 | 0 | 0 | 0 | 0 | 0 | 0 |
| 6 | 0 | 0 | 0 | 0 | 0 | 1 | 0 | 0 |
| 7 | 0 | 0 | 0 | 0 | 0 | 1 | 0 | 0 |
| 8 | 0 | 0 | 1 | 0 | 0 | 1 | 0 | 0 |
| 9 | 0 | 0 | 1 | 0 | 0 | 1 | 0 | 0 |
| 10* | 0 | 0 | 1 | 0 | 0 | 1 | 0 | 0 |
| 11 | 0 | 0 | 1 | 0 | 0 | 1 | 0 | 0 |
| 12 | 0 | 0 | 1 | 0 | 0 | 1 | 0 | 0 |
| 13 | 0 | 0 | 1 | 0 | 0 | 1 | 0 | 0 |
| 1= Significant difference | | | | | | | | |
